# Efficacy of Mindfulness-Based Cognitive Therapy in the Recovery Phase of Schizophrenia

**DOI:** 10.1192/j.eurpsy.2026.10914

**Published:** 2026-07-31

**Authors:** H. C. Zhang

**Affiliations:** The 988th Hospital of the People’s Liberation Army, Jiaozuo City, Henan Province, China

## Abstract

**Introduction:**

Background: Individuals in the recovery phase of schizophrenia often face persistent challenges including residual symptoms, emotional dysregulation, cognitive deficits, and high relapse rates. Mindfulness-Based Cognitive Therapy (MBCT), effective in mood disorders, shows promise as an adjunctive intervention but requires rigorous evaluation in schizophrenia recovery.Background: Individuals in the recovery phase of schizophrenia often face persistent challenges including residual symptoms, emotional dysregulation, cognitive deficits, and high relapse rates. Mindfulness-Based Cognitive Therapy (MBCT), effective in mood disorders, shows promise as an adjunctive intervention but requires rigorous evaluation in schizophrenia recovery.

**Objectives:**

This study examined the efficacy of MBCT in improving psychological functioning and reducing relapse risk among individuals with schizophrenia during the recovery phase.

**Methods:**

A randomized controlled trial (RCT) was conducted with 80 clinically stable outpatients diagnosed with schizophrenia. Participants were assigned to either an 8-week MBCT program plus treatment-as-usual (TAU) (n=40) or TAU alone (n=40). Primary outcomes included relapse rates (measured over 12 months), symptom severity (PANSS, SANS), and mindfulness skills (FFMQ). Secondary outcomes encompassed depression/anxiety (HADS), cognitive functioning (MoCA), and quality of life (SQLS). Assessments occurred at baseline, post-intervention, and 6/12-month follow-ups.

**Results:**

The MBCT group demonstrated significantly lower relapse rates (15% vs. 37.5%, p<0.01) and greater reductions in negative symptoms (SANS: p=0.003) and emotional distress (HADS-depression: p=0.008) compared to TAU at 12 months. Significant improvements in mindfulness skills (FFMQ-Observe, p<0.001; FFMQ-Nonreact, p=0.002), attention regulation (MoCA, p=0.015), and quality of life (SQLS, p=0.004) were also observed post-intervention and sustained at follow-up. No adverse events were reported.

**Conclusions:**

MBCT is a feasible and effective adjunctive therapy for individuals recovering from schizophrenia, enhancing mindfulness, mitigating negative symptoms and emotional distress, improving cognitive function, and reducing relapse risk. Integration of MBCT into recovery-phase care may promote long-term stability and functional outcomes.

**Disclosure of Interest:**

None Declared

